# Modern Sociology

**Published:** 1903-11-07

**Authors:** 


### Modern Sociology.

### DETERIORATION OF THE BREED.

A MATHEMATICAL proof of what is common belief may not seem a very urgent matter, and yet there are statistical demonstrations of the obvious that have their uses. It is familiar knowledge to most persons observant of their neighbours, that mental and moral qualities, as well as physical, are apt to run in families, or that they are matters of breeding. On the other hand, so much has been said of the influence of environment, and of the power of education, that it has been thought desirable to put the question to an exact test. Professor Karl Pearson, a mathematician who applies the calculus to data of highly human interest, has lately, in the form of the Huxley Memorial Lecture at the Anthropological Institute, given the results of a 7-years' inquiry upon the inheritance of physique and health, of intelligence, and of moral qualities. For the question of inherited physical characters, the method was to take certain measurements in more than a thousand families. For the much more subtle question of mental and moral inheritance, the method was to take the opinions of teachers in 200 schools of every kind throughout the country. It need not be said that the labour has been very great, and that it has involved the zealous co-operation of many. The matters of fact are not all equally ascertainable. The ordinary anthropometric data admit of a good deal of precision; but in the same class is included "general health," which can only be a matter of rough guessing. Among mental qualities, intelligence, or quickness of apprehension, can be estimated by most teachers, by some better than by others; but memory is not so easy to measure, and imagination is probably impossible altogether. However, the data were ascertained somehow, and subjected to the calculus. Brother having been compared with brother, sister with sister, and brother with sister, the result came out, for physical characters, that if one of the pair exceeded or fell short of the mean by a certain amount, the other of the pair tended to exceed or fall short of the mean by half that amount. The general result of the inquiry into mental and moral characters was to show that a similar relation existed in the members of a family. The general health in the community was inferred to be inherited quite as much as the cephalic index and other measurable bodily things. Mental and moral qualities had also a constant relation to the physical heredity. Physique, health, intelligence, and character were found to be all alike determined by the breed or stock. Home influences, education, and whatever else may come under environment will foster but not create; and "the relative gain from education" depends to a surprising degree upon the quality of the raw material.

As this result of a highly complex statistical inquiry tends to confirm what has been commonly believed and has even passed into our proverbs, maxims and prejudices, the data and methods by which it was reached are all the more entitled to credit. There being no question then, as to the indispensableness of a good stock to breed from, the practical questions which confront us take a very definite shape. The physician of the body politic, making heredity his ground principle, comes next to the diagnosis of the malady or alleged malady. Professor Pearson does not doubt that there is deterioration. He is bold to assert that there has been a falling off during the last forty years in the intelligence of the British merchant, workman, and professional man; and he believes that the cause of it has been that the better stocks in the community have not been reproducing in due proportion to the less able and less capable, which are the more prolific. The intellectual classes have become enervated by wealth or by love of pleasure, or by following erroneous ideals, and are not furnishing their quota of the men who are needed to carry on the ever-growing work of the Empire. As to the remedy, he tells us what it should not be, and leaves us to infer what it should be; it will not help us to copy foreign methods of instruction or to extend technical education. At this interesting point the lecture ends, its real object having been to prove the all-importance of a good breed or stock for the national physique, health, intelligence and character. Without seeking to speculate beyond the lecturer's own limit, one cannot but recall what Huxley himself, in whose memory the lecture was held, had to say on one or more occasions upon this same question of holding our own among the nations of the globe. He was speaking at a dinner of a City company which had just admitted him to its membership, and he complimented them, as was only right and proper, upon having given some of their wealth to help the Technical Institute, which was then being started. But the burden of his speech was something else. He spoke of the strong and original thinkers who had been, in the end of the eighteenth century, the pioneers of our unique industrial progress, not only the chemists and physicists, but also the political economists; and he declared that, without a succession of such men, holding a firm grasp of principles, our national supremacy would decline, and we should share the fate of the great empires of antiquity.

This is more the point of view of Carlyle than of anthropology, the question of getting and keeping the genius or the hero rather than of keeping up the stock of the "intellectual classes," as Professor Pearson is reported to have named them. But the difference is probably more than in the point of view. Who are the intellectual classes? And what reason is there to suppose that this superior part of the nation would keep up the succession of ability and efficiency within its own limits even if it were as prolific as the rest? Ability has sprung in many instances from a stock which would hardly have been pronounced intellectual, whatever other qualities it had. The highest degree of ability, or original genius, has sprung still more frequently from an ancestry which could hardly have been suspected to contain the germs. A theory of that kind of heredity was once propounded by Renan, which has a very important practical bearing if it be correct. Speaking amongst his own Breton kinsfolk, in the town where his statue has lately been set up, he declared that whatever mental endowment he had was owing to the mental savings or intellectual parsimony of his obscure and undistinguished ancestors, who had lived well within themselves and made as it were a reserve of unused intelligence to transmit in the breed. It would take some time to elucidate so bold a paradox, which seems to contradict all our maxims about increasing the faculties by exercising them. Have we not the authority of Hamlet that our capability and God-like reason will "fust" in us if they are unused? Renan appears to have reflected that, in contrast with the lives of those old-world Bretons, there may be such a thing as living upon intellectual capital, to the impoverishment of the stock. To say that our intellectual classes are enervated by wealth, luxury and the like, hardly covers this point. Would it not be more to the point to say that many of them are not sufficiently endowed with the unused intelligence of their mothers to start with, and that most of them suffer throughout life from the effects of incessant competitive and pass examinations? In the latter respect, at least, the Germans have had a certain advantage of us—they have been wonderfully thrifty in the use of examinations.

